# Impact of Nanonutrient Complex on Growth Performance, Feed Efficiency, and Hematobiochemical Profiles in Asian Catfish, *Clarias batrachus*

**DOI:** 10.1155/anu/7382715

**Published:** 2025-08-24

**Authors:** Md. Ayenuddin Haque, Md. Abu Sayed Jewel, Nasrin Akter, Sumaiya Akter, Arun Chandra Barman, S. M. Nurul Amin, Takaomi Arai, Mohammed Fahad Albeshr, Norhayati Ngah, Mohammad Belal Hossain

**Affiliations:** ^1^Department of Oceanography and Blue Economy, Faculty of Fisheries, Habiganj Agricultural University, Habiganj 3300, Bangladesh; ^2^Department of Fisheries, Faculty of Agriculture, University of Rajshahi, Rajshahi 6205, Bangladesh; ^3^Curtin Aquatic Research Laboratories, School of Molecular and Life Science, Faculty of Science and Engineering, Curtin University, Perth, Western Australia 6102, Australia; ^4^Environmental and Life Sciences Programme, Faculty of Science, Universiti Brunei Darussalam, Jalan Tungku Link, Gadong, BE 1410, Brunei Darussalam; ^5^Department of Zoology, College of Science, King Saud University, P.O. Box 2455, Riyadh 11451, Saudi Arabia; ^6^East Coast Environmental Research Institute, Universiti Sultan Zainal Abidin, Gong Badak Campus 21300, Kuala Nerus, Terengganu Darul Iman, Malaysia; ^7^School of Engineering and Built Environment, Griffith University, Nathan Campus, Brisbane, QLD, Australia; ^8^Department of Fisheries and Marine Science, Noakhali Science and Technology University, Noakhali 3814, Bangladesh

**Keywords:** aquafeed innovation, blood indices, feed supplementation, growth optimization, immunity, nanoparticle-enriched diet, serum enzymes, sustainable aquaculture, toxicity threshold

## Abstract

Understanding how nanonutrients influence the growth and physiological processes of cultivable fish can boost fish production efficiency with less management, advancing aquaculture toward global food security. In this study, a 60-day feeding trial was conducted to determine the effects of a nanonutrient complex (NNC) on the growth performances and physiology of Asian catfish, *Clarias batrachus*. Nanoparticles (NPs; Zn, Cu, and Fe) were synthesized from their metallic salts using an established acoustic method and characterized via scanning electron microscopy. The NNC was formulated by mixing zinc NPs (Zn-NPs), copper NPs (Cu-NPs), and iron NPs (Fe-NPs) in a 40:20:40 ratio. In the experiment, a basal diet was supplemented with NNC at concentrations of 0.0 (control), 10, 20, 30, 40, and 50 mg/kg and fed to *C. batrachus* for 60 days to evaluate growth and physiological parameters (hematology, lipid, and enzyme profiles). The findings indicated that fish administered 30 mg/kg NNC achieved the highest final weight (FW; 25.73 ± 0.41 g), weight gain (WG; 386.67% ± 10.12%), average daily gain (ADG; 0.34 ± 0.01 g/fish/day), specific growth rate (SGR; 2.64 ± 0.03%/day), and enhanced feed conversion ratio (FCR; 1.24 ± 0.03), with statistically significant differences (*p*  < 0.05) relative to the control group. Regression study determined the ideal NNC dosage range to be 30.19–30.26 mg/kg for growth and FCR results. Muscle composition enhanced at this level, with protein and fat content attaining 18.6% ± 0.3% and 6.8% ± 0.2%, respectively. Hematological indices reached their optimum at 30 mg/kg NNC, with red blood cell (RBC) count (3.62 ± 0.21 × 10^6^/mm^3^), hemoglobin (Hb; 9.83 ± 0.12 g/dL), and hematocrit (Hct; 26.31% ± 0.52%) greatly surpassing those of other treatments. Serum biochemical analysis indicated elevated total protein (4.79 ± 0.05 g/dL), albumin (1.55 ± 0.04 g/dL), and globulin (3.24 ± 0.02 g/dL) at 30 mg/kg, while undesirable elevations in stress markers—cholesterol (224.84 ± 1.10 mg/dL), alanine aminotransferase (ALT; 33.75 ± 0.39 U/L), and aspartate aminotransferase (AST; 40.29 ± 1.17 U/L)—were noted at 50 mg/kg, suggesting potential toxicity at increased dosages. Bioaccumulation of trace elements was most pronounced in the liver, with copper concentrations 11.15% greater than zinc and 1.09% higher than iron, while overall accumulation in the liver surpassed that in muscle and serum by 21.84% and 57.84%, respectively.

## 1. Introduction

Fish production in aquaculture is significantly impacted by both diet and water quality, which are critical for determining profitability. Feed costs account for approximately 56.45%–58.49% of total production expenses, sometimes reaching as high as 70%, making high-quality feed production a challenge, particularly for small-scale farmers [1–3]. Degraded water quality, characterized by the accumulation of harmful chemicals like arsenic, ammonia, nitrite, hydrogen sulfide, and the presence of pathogens, also poses severe risks to fish health and productivity [4]. In response to these challenges, nanoparticles (NPs) offer a promising solution by improving nutrient delivery, enhancing feed efficiency, and supporting sustainable aquaculture practices [5, 6]. The use of nanotechnology in feed production not only improves fish growth and health but also addresses water quality concerns, offering an economically viable and ecologically sustainable approach to aquaculture.

Nanotechnology plays a crucial role in aquaculture feed production, effectively delivering micronutrients and enhancing the growth of aquatic organisms [7]. Nanonutrient supplementation improves bioavailability, absorption, utilization, and cellular processes [8]. The application of NPs notably boosts digestive enzyme activation, increases villi length and breadth, and enhances the number and integrity of goblet cells, thereby improving feed utilization and metabolic function [9]. For example, Kumar et al. [10] demonstrated that feeding *Pangasianodon hypophthalmus* with 15 mg kg^−1^ of iron NPs (Fe-NPs) led to the upregulation of growth hormone regulators (GHR1 and GHRb), growth hormone (GH), and insulin-like growth factors (IGFs 1x and IGF 2x). Additionally, Fe-NPs have been shown to lower levels of alanine aminotransferase (ALT) and aspartate aminotransferase (AST), thereby improving fish health [11]. Furthermore, Kumar et al. [12] reported that zinc NPs (Zn-NPs) activated antioxidant defense systems, enhancing the activity of superoxide dismutase (SOD), glutathione reductase (GR), catalase (CAT), glutathione-S-transferase (GST), and glutathione peroxidase (GPx) in *P. hypophthalmus* fed with 10 and 20 mg kg^−1^ of NPs.

Metal-based NPs such as iron, copper, zinc, and selenium show significant potential in aquaculture and fish feed technology, enhancing growth, feeding efficiency, and cost-effectiveness [13–15]. Zn-NPs are particularly noted for their non-toxicity, biosafety, and heat resistance, improving growth and feed efficiency in species like *Clarias batrachus*, *Labeo rohita*, and *Oreochromis niloticus* [15–18]. Additionally, Cu-NPs have been shown to enhance growth performance in *C. batrachus* and common carp [14, 19], while iron Fe-NPs improve the physiology of *C. batrachus*, *L. rohita*, and *O. niloticus* [20–22]. However, caution is warranted as excessive use of metal-based NPs can lead to acute toxicity and decreased growth performance, immunity, and increased genotoxicity [23, 24]. While concurrent administration of various NPs shows promise for promoting growth and antioxidative status in multiple fish species, further research is needed to explore the combined effects of Zn, Cu, and Fe NPs in fish diets.

The Asian catfish (*Clarias batrachus*, Linnaeus, 1758) is widely distributed in freshwater ecosystems throughout South and Southeast Asia, inhabiting diverse environments such as rivers, lakes, and floodplains. Its ability to thrive in stressful conditions, including low oxygen levels, along with rapid growth rates, makes it an appealing option for aquaculture [25, 26]. This species is highly valued for its flavor and nutritional quality, playing a crucial role in local economies and food security [27, 28]. However, the culture of Asian catfish in Bangladesh and elsewhere face increasing challenges due to declining water quality from pollution and nutrient runoff, rising feed costs, and additional stressors from climate change, such as altered temperature and precipitation patterns, which jeopardize fish production, fish health, and the livelihoods of local farmers. Therefore, integrating nanonutrients into fish diets and investigating their effects on growth rate and hematological profiles can provide critical insights into fish health and stress responses, which are vital for developing sustainable aquaculture practices in the context of declining water quality and increasing pollution levels. Notably, such research has not yet been conducted in Bangladesh, highlighting a significant knowledge gap in the application of nanotechnology within the country's aquaculture sector. This study assessed the impact of a nanonutrient complex (NNC) containing Zn-NPs, Cu-NPs, and Fe-NPs on the growth performance, nutrient absorption, and hematological profiles of *C. batrachus*. The primary aim was to determine the optimal dosage of NNC to improve growth and physiological functions in this economically significant species. The findings could lead to innovative dietary strategies that promote fish growth, welfare, and resilience, ultimately benefiting local fisheries and communities dependent on this vital species.

## 2. Materials and Methods

### 2.1. Ethical Approval

This study was approved by the guidelines and regulations of Department of Fisheries, University of Rajshahi, Bangladesh.

### 2.2. Synthesizing and Characterization of NPs

NPs were synthesized utilizing a modified method of Alam et al. [29] using the acoustic method. Zinc acetate ([Zn(CHCOO)_2_·2HO], 99.99%), cupric acetate monohydrate [(CH_3_COO)_2_Cu·H_2_O], FeSO_4_·7H_2_O, and ethylene glycol (EG, 322 99.8%) were obtained from Sigma–Aldrich (Merck KGaA, Darmstadt, Germany). Polyvinylpyrrolidone (PVP, M. Wt. 130,000) was purchased from AcrosOrganics BVBA (Belgium) and used separately to synthesize Zn-NPs, Cu-NPs, and Fe-NPs. The NNC was formulated by mixing Zn-NPs, Cu-NPs, and Fe-NPs in a 40:20:40 ratio. This ratio was selected based on empirical optimization from preliminary dose–response trials and documented nutritional requirements for similar fish species, particularly *Clarias batrachus* [6, 14, 30–34]. The results demonstrated that elevated concentrations of Cu-NPs exhibited a more restricted safety margin relative to Zn and Fe. Consequently, a comparatively lesser proportion (20%) of Cu-NPs was utilized to mitigate potential pro-oxidant effects, although Zn and Fe, possessing broader tolerance ranges, were employed at 40% each to guarantee sufficiency without exceeding safe intake thresholds. Metallic salts, EG (322 99.8%), and PVP (M. Wt. 130,000) were combined in 25 mL water and heated at 70°C for 45 min in oil bath heater (Oil Bath Digital (13L) SH-WB-10GDH SH Scientific Korea). Nanostructures of particular shapes and sizes were synthesized in large numbers by employing experimental features like reagent surfactant concentrations (such as PVP), solvent types and amounts (EG or other polyols), gas bubbling, temperature, and heating rate [29].

The synthesized NPs were purified using the precipitation process. The crystal structures were studied employing an atomic force microscope (AFM; Park Systems, XE-70, South Korea). The nanosolution was centrifuged for three times at 6000 revolutions per minute (rpm; Centrifuge Machine 6800 rpm 8 × 15 mL RB/4 × 15 mL Tube Z 207A HERMLE Germany), each centrifugation lasting 30 min. This procedure was carried out to ensure that all products were thoroughly extracted during centrifugation. Particles were gathered and then introduced into ethanol to disperse. AFM was measured using droplets of colloidal solution on glass slides. A spectrophotometer (Varian Cary 6000i, Santa Clara, CA, USA) characterized the NPs at 300–500 nm, revealing a peak of 260–380 nm [15]. Produced Fe-NPs were reddish-brown in color, crystalline, quasi-spherical in shape and their average size and zeta potential were ranged from 75.0 to 120.45 nm and 50.5 ± 1.0 mV. Cu-NPs were roughly spherical in shape, 85.0 nm in size and zeta potential was 43.0 ± 1.0 mV. Furthermore, Zn-NPs were mostly needle-shaped nanorods with mean size of 14.7 ± 2.5 nm. The zeta potential of Zn-NPs was −32.5 ± 1.0 mV.

### 2.3. Preparation of Basal Diet

The basal diet was prepared from locally sourced feed items such as fish meal, mustard oil cake, soybean meal, maize bran, wheat bran, rice bran, and soybean oil. The feed was supplemented with zinc, copper, and iron-free minerals, as well as a vitamin premixture (Table 1). The ingredients selected went through a variety of processes such as grinding, sifting, weighing, and thorough blending. The basal diet was supplemented with synthesized NNC at five dosages (10, 20, 30, 40, and 50 mg/kg). Selected doses of NNC were combined with the the basal diet and mixed for 5 min. A sufficient amount of water was added to obtain the desired dough-like texture in the final mixture. The dough was pelletized using a manual pelletiser of 3 mm diameter dies. Pellets were gathered and placed on an aluminum plate before drying in a thermostatic hot air oven (Microsil, India). The drying procedure helps to keep moisture levels below 10%. Dried feeds were stored at −20°C until used in the experiment.

### 2.4. Experimental Design

**Figure 1:**
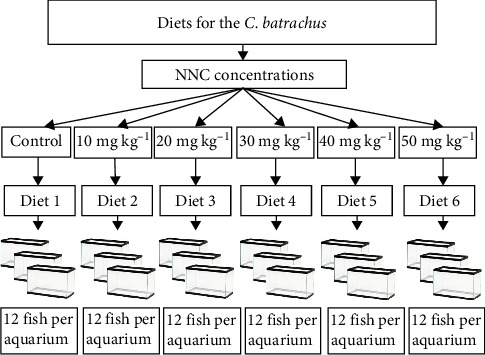
Schematic layout of the experimental design. Six treatments were administered (Control, 10, 20, 30, 40, and 50 mg/kg NNC), with three replicate tanks per treatment and 12 *Clarias batrachus* per tank.

A total of 216 fish, with an average weight of 5.23 ± 0.06 g (mean ± SEM), were obtained from a commercial hatchery and transported to the laboratory in aerated plastic bags. In the laboratory, fish were fed with the basal diet for 7 days in concrete tank with a steady water flow. After the acclimatization, fish were evenly distributed in 18 glass tank (12 fish/tank). The tankss were 1.5 m × 0.8 m × 0.8 m in dimension and having a capacity of 500 L (Figure 1). The 18 aquariums were grouped into six treatments, considering three aquariums for each treatment based on the concentration of NNC in feed and basal diet as control. The feeding study lasted 60 days and the fish were hand fed twice daily at a rate of 3% of their body weight. After 5 h of feeding, all the remaining residual feed particles and feces were siphoned regularly. During the culture period, each tank underwent a daily water exchange in which about 50% of water was changed. Periodic evaluation of physicochemical parameters (temperature, °C; dissolved oxygen (DO), mg/L, pH, and ammonia, mg/L) was performed fortnightly using a Celsius thermometer for temperature, dissloved oxygen by a HACH kit (FF-2, USA) and pH by a pH meter (Jenwary 3020). The value of these parameters were 26.71–27.96°C, 5.27–5.87 mg/L, 7.00–7.26, and 0.001–0.002 mg/L, respectively.

### 2.5. Growth Parameters and Feed Utilization

Fish were sampled fortnightly and measured for total length and weight. Total body weight and length were measured to the nearest g and cm, with 0.1 g and 0.1 cm accuracy using an electronic balance and digital slide caliper. The fish were collected and measured for final biomass at the end of the feeding session. Fish growth parameters, condition factor (CF), survival rate (SR), and feed utilization parameters were individually calculated by the following equations listed below [35]:

(1) $$\begin{array}{c} \%\text{Weight gain }\left(\text{g}\right)=\frac{\text{Final weight}\left(g\right)-\text{initial weight (g)}}{\text{Initial weight (g)}}\times 100. \end{array}$$

(2) $$\begin{array}{c} \text{Average daily gain, ADG (g/fish/day)}=\frac{\text{Final weight (g)}-\text{initial weight (g)}}{\text{Period of feeding trial (days)}}. \end{array}$$

(3) $$\begin{array}{c} \text{Specific growth rate, SGR (\% bw./day)}=\frac{\text{ln Final weight}-\text{ln initial weight}}{\text{Experimental period }\left(\text{days}\right)}\times 100. \end{array}$$

(4) $$\begin{array}{c} \text{Condition factor, CF}=\frac{\text{Body weight (g)}}{{\text{Body length (cm)}}^{3}}\times 100. \end{array}$$

(5) $$\begin{array}{c} \text{Survival rate, SR (\%)}=\frac{\text{No. of fish survived}}{\text{Initial no}.\text{ of fish stocked}}\times 100. \end{array}$$

(6) $$\begin{array}{c} \text{Feed conversion ratio, FCR}=\frac{\text{Total feed intake (g)}}{\text{Total weight gain (g)}}. \end{array}$$

(7) $$\begin{array}{c} \text{Protein efficiency ratio, PER}=\frac{\text{Total weight gain (g)}}{\text{Total protein intake (g)}}. \end{array}$$

(8) $$\begin{array}{c} \text{Protein growth rate, PGR (\%)}=\frac{\text{ln Final protein}-\text{ln initial protein}}{\text{Experimental period (days)}}\times 100. \end{array}$$

(9) $$\begin{array}{c} \text{Annual net protein unilization, ANPU (\%)}=\frac{\text{Net increase}\in \text{carcass protein}}{\text{Protein consumed}}\times 100. \end{array}$$

### 2.6. Proximate Composition of Feed and Fish Muscle

At the ending of the feeding experiment, basal diet and muscle from 15 randomly selected specimen from each treatment were collected and examined for protein, lipid, carbohydrate, ash, and moisture according to AOAC [36]. The samples were tested both the start and end of the study. The Kjeldahl method (JEQ-16B, India) measured crude protein, whereas the Soxhlet extraction method (LABORATE, LT-SOXA001, India) measured crude lipids. The ash content was determined by heating the sample to a muffle furnace (SH-FU-5MG SH Scientific Korea) operating at 550°C for 5 h. The relative humidity was measured by drying the sample in the oven at 70 and 105°C.

### 2.7. Hematological Parameters

To obtain blood samples, 15 randomly chosen fish from each feeding group were anesthetized with 50 µL of clove oil (Merck, Germany) in one liter1 L of water. Fish blood samples were collected from the caudal vein using a 2.0 mL hypodermal syringe. Two vials were rapidly filled with approximately 500–600 µl of blood. One vial was treated with EDTA to prevent anticoagulation of the blood while serum sample was collected in other vial without any anticoagulant. The vials containing a coating of EDTA were subjected to gentle agitation to prevent the incidence of hemolysis and blood clotting. The collected blood samples were subsequently utilized for laboratory analysis. After diluting the blood samples with Turk's and Toisson's solutions, a Neubauer hemocytometer was used to count the cells [21, 37]. The total number of red blood cells (RBCs) per milliliter is equal to 20,050 *N* and 10,000 *N* (where *N* is the number of RBCs measured and dilution factor is 200). The total number of white blood cells (WBCs) per milliliter is equal to (201 *L*) (0.4) cells 50 L (where *L* is the number of WBCs counted and dilution factor is 50). Blood hemoglobin (Hb) was measured using the cyanomethemoglobin technique and Drabkin's solution [38] and hematocrit (Hct) using the usual micro-Hct method [39]. Micro-Hct tubes were used to assess erythrocyte MCV, MCH, and MCHC [40].

### 2.8. Serum Biochemical Parameters

Blood samples taken without anticoagulants were utilized for serum analysis. To separate the serum from the blood samples, the tubes were put in a vertical position for 2 h. Each tube was subjected to centrifugation at 2000 rpm for 15 min at 4°C. The serum samples were collected using a micropipette and then kept at −20°C until suitable for use. Total protein and albumin were analyzed spectrophotometry and globulin was determined by subtracting albumin from complete protein. Total cholesterol, triglycerides, high-density lipoprotein (HDL), low-density lipoprotein (LDL), ALT, AST, and alkaline phosphatase (ALP) levels were measured using commercially available Crest Biosystems atomic absorption spectrophotometry kits. Serum enzyme activity was colorimetrically measured to determine amylase, lipase, and protease levels.

### 2.9. Bioaccumulation of Trace Elements

The muscle, liver, and serum samples obtained from the experimental fish were used for a bioaccumulation study. The fish specimen used for blood sample collection was dissected using surgical scissors to collect the liver and muscles. The liver and muscles were sampled and cleansed with double distilled water before being stored in 10% formalin for further study. Trace elements (Zn, Cu, and Fe) were quantified in liver, muscle, and serum samples using inductively coupled plasma mass spectrometry (ICP-MS) [41]. A 1 g of freeze-dried sample was treated with 10 mL of concentrated nitric acid and 2 mL of perchloric acid. The digesting step included heating the solution on a heater set at 100°C. The samples were treated with hydrogen peroxide (two drops) and then, filtered with Whatman filter paper. Content on trace elements in the fish muscle was measured in µg/g.

### 2.10. Statistical Analysis

The results were reported as mean ± S.E and the normality was assessed using the Shapiro–Wilk test. Before analysis, percent and ratio data were arc-sign converted. The influence of different NNC concentration on growth parameters, feed utilization, hematology, and lipid and enzymatic profile of serum was investigated using a one-way analysis of variance (ANOVA) using SPSS 25 version. Statistical significance were measured at a 5% probability level using Duncan's multiple range test (DMRT).

## 3. Results

### 3.1. Water Quality Parameters

Water quality measurements (Table 2) consistently fell within optimum levels throughout all treatments, with no significant variations (*p*  > 0.05). Temperature varied from 27.89 ± 0.30 to 28.75 ± 0.22°C, DO from 5.07 ± 0.17 to 5.44 ± 0.33 mg/L, pH from 7.00 to 7.50, while ammonia concentrations were consistently low (0.001–0.003 mg/L), signifying a nonstressful environment. Futher, no adverse impacts of NNC supplementation on water quality were seen in the experimental aquarium (Table 2).

### 3.2. Growth Performance

**Figure 2:**
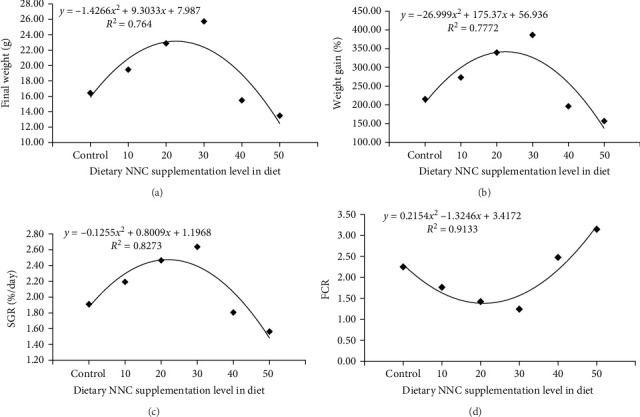
The regression analyses showing the relationship between dietary NNC levels and (A) final weight (FW), (B) percentage weight gain (%WG), (C) specific growth rate (SGR; %/day), and (D) feed conversion ratio (FCR) in *Clarias batrachus* after 60 days of feeding. Each point represents mean ± SEM.

Substantial enhancements in growth performance were recorded in *Clarias batrachus* administered diets with 30 mg/kg NNC than the control group (Table 3). This group attained the maximum final weight (FW; 25.73 ± 0.41 g), weight gain percentage (%WG; 386.67% ± 10.12%), average daily gain (ADG; 0.34 ± 0.01 g/fish/day), and specific growth rate (SGR; 2.64 ± 0.03%/day). The feed conversion ratio (FCR) was minimal (1.24 ± 0.03), but the protein efficiency ratio (PER) was maximal (2.44 ± 0.06) at this level. The control group demonstrated a FW of 16.43 ± 1.33 g, a WG of 215.33% ± 25.77%, a SGR of 1.91% ± 0.13% per day, and a FCR of 2.25 ± 0.26. Fish fed with 30 mg/kg NNC group gave 36% higher FW than the control group. In contrast to the control group, the growth performance significantly decreased when the dietary NNC content was further elevated to NNC40 or NNC50. According to the second-order polynomial regression between dietary NNC levels and FW (g), WG%, and SGR (%/day), the ideal nutritional NNC level for *C. batrachus* was ranged from 30.19 and 30.26 mg/kg (Figure 2). The fish in all groups exhibited good health, as indicated by the absence of any mortality (*p*  > 0.05) throughout the experiment.

### 3.3. Feed Utilization Parameters

The FCR demonstrated a noticeable decrease pattern, particularly at levels of up to 30 mg/kg NNC compared to the control group (Table 3). The FCR was markedly lower in the NNC30 group (1.24 ± 0.03) compared to the control group (2.25 ± 0.26), signifying improved feed efficiency. The PER and annual net protein utilization (ANPU) were highest in NNC30, measured at 2.44 ± 0.06 and 39.70% ± 3.03%, respectively, compared to 1.36 ± 0.16 and 21.32% ± 1.90% in the control group. The protein growth rate (PGR) reached its zenith in the NNC40 group at 1.69% ± 0.10%, slightly surpassing the 1.60% ± 0.10% observed in NNC30 and the 1.37% ± 0.01% recorded in the control group. According to the second-order polynomial regression between dietary NNC levels and FCR, the ideal dietary NNC level for *C. batrachus* was 30.07 mg/kg (Figure 1). Except for PGR (%), which showed its highest value at 40 mg/kg NNC in diet, the PER and ANPU (%) trends were contrary to FCR's.

### 3.4. Proximate Composition

**Figure 3:**
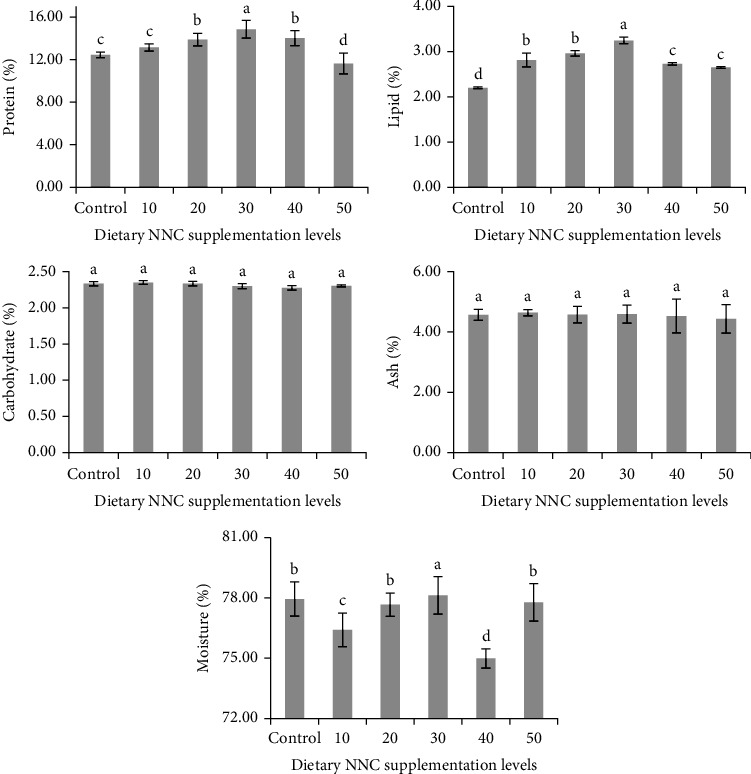
Proximate composition (mean ± SEM) of muscle tissue from *Clarias batrachus* fed varying levels of nanonutrient complex (NNC) in the diet. Bars with different superscript letters indicate significant differences (*p*  < 0.05) among treatments for each nutrient component.

A significant difference (*p*  < 0.05) was seen in muscle protein, lipid, and moisture content between NNC and control groups (Figure 3). The NNC30 group exhibited maximum crude protein and lipid content at 18.6% ± 0.3% and 6.8% ± 0.2%, respectively, in contrast to 16.1% ± 0.3% and 4.8% ± 0.2% seen in the control group. The moisture content in NNC30 (74.2% ± 0.2%) was markedly lower than in the control (76.4% ± 0.5%), suggesting enhanced nutrient deposition. NNC20 exhibited increased protein (18.1% ± 0.4%) and lipid (6.2% ± 0.2%) concentrations; however, subsequent increments in dietary NNC (40–50 mg/kg) resulted in a reduction in proximate composition. Fish administered 50 mg/kg displayed the lowest protein (16.0% ± 0.3%) and lipid (4.1% ± 0.2%) concentrations.

### 3.5. Hematological Parameters

Dietary supplementation with a NNC markedly improved the hematological profiles in *Clarias batrachus* (Table 4). The RBC count, Hb concentration, and Hct exhibited a progressive increase with escalating NNC levels up to 30 mg/kg, attaining peak values in the NNC30 group (3.62 ± 0.21 × 10^6^/mm^3^, 9.83 ± 0.12 g/dL, and 26.31 ± 0.52%, respectively), in contrast to the control group (2.88 ± 0.02 × 10^6^/mm^3^, 9.31 ± 0.10 g/dL, and 25.17% ± 0.92%). At elevated supplementation levels (NNC40 and NNC50), these values significantly decreased, with RBC falling to 2.31 ± 0.14 × 10^6^/mm^3^, Hb to 6.82 ± 0.62 g/dL, and Hct to 17.85% ± 0.84% in NNC50. The WBC count exhibited a substantial rise in a dose-dependent manner across treatments, escalating from 6.69 ± 0.22 × 10^6^/mm^3^ in the control group to 8.77 ± 0.34 × 10^6^/mm^3^ in the NNC50 group, suggesting potential immunological stress at elevated NNC concentrations. The erythrocyte indices exhibited a same trend. The mean MCV and MCH attained peak values in the NNC30 group (87.52 ± 3.62 fL and 32.46 ± 1.21 pg/cell, respectively), greatly surpassing those of the control group (72.86 ± 4.96 fL and 27.19 ± 1.24 pg/cell). MCHC levels exhibited no statistically significant variation between treatments (*p*  > 0.05), ranging from 3.65% ± 0.11% to 4.09% ± 0.27% supplementation.

### 3.6. Serum Biochemical Profile

The serum biochemical parameters assessed in both the experimental and control groups are shown in Table 5. The total protein, albumin, and globulin concentrations were maximized in fish with 50 mg/kg NNC, measuring 4.95 ± 0.05 g/dL, 1.59 ± 0.06 g/dL, and 3.97 ± 0.04 g/dL, respectively, in contrast to 3.14 ± 0.06 g/dL, 0.47 ± 0.02 g/dL, and 2.66 ± 0.04 g/dL in the control group. In the 30 mg/kg NNC group, the values recorded were 4.79 ± 0.05 g/dL for total protein, 1.55 ± 0.04 g/dL for albumin, and 3.24 ± 0.02 g/dL for globulin. Total cholesterol in the NNC30 group reduced to 193.71 ± 4.87 mg/dL, compared to 212.72 ± 2.48 mg/dL in the control group, but it climbed to 224.84 ± 1.10 mg/dL in the NNC50 group. HDL levels varied from 52.28 ± 0.34 mg/dL (NNC30) to 55.75 ± 1.13 mg/dL (NNC50), whilst LDL levels rose from 150.99 ± 1.23 mg/dL (control) to 154.83 ± 0.82 mg/dL (NNC50). Triglyceride levels were 138.53 ± 3.36 mg/dL in the control group and 159.80 ± 3.07 mg/dL in the NNC50 group. ALP levels rose from 7.85 ± 1.03 mg/dL (control) to 17.08 ± 2.37 mg/dL (NNC50). ALT and AST levels peaked at 50 mg/kg NNC, at 33.75 ± 0.39 U/L and 40.29 ± 1.17 U/L, respectively, in contrast to 28.47 ± 0.85 U/L and 34.04 ± 0.50 U/L in the control group. The activities of digestive enzymes (U/L) likewise increased with the intake of NNC. Amylase increased from 0.31 ± 0.02 (control) to 1.80 ± 0.11 (NNC50), lipase from 0.27 ± 0.03 to 1.85 ± 0.07, and protease from 0.66 ± 0.04 to 1.51 ± 0.06.

### 3.7. Concentration of Zn, Cu, and Fe in Muscle, Serum, and Liver

**Figure 4:**
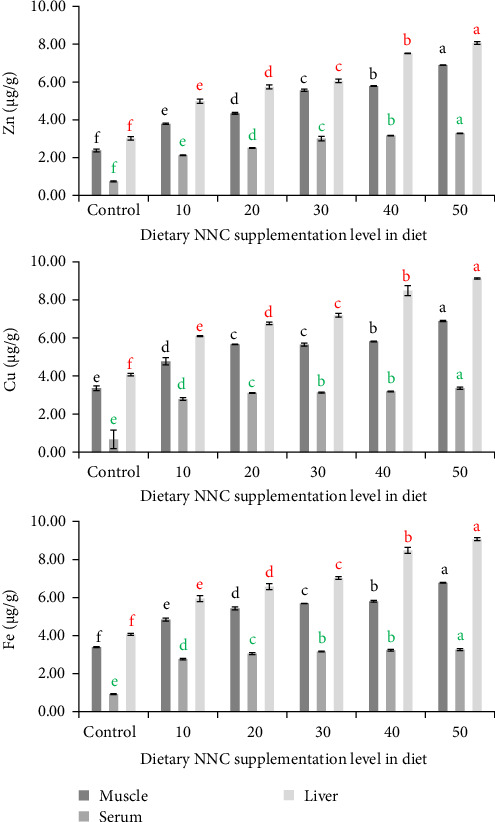
Concentrations of Zn, Cu, and Fe in muscle, serum, and liver of *Clarias batrachus* fed on different levels of NNC in diet for 60 days. Different alphabet on top of each bar indicate significant difference in Zn, Cu, and Fe content of muscle (black), serum (green), and liver (red) among the dietary NNC supplementation level in diet.

Concentrations of trace elements in the muscle, serum, and liver of *Clarias batrachus* exhibited considerable variation among different treatments (Figure 4). The liver consistently had the highest concentrations of Zn, Cu, and Fe across all tissues, followed by muscle and serum. The average metal concentrations in the liver exceeded those in muscle by 21.84% and surpassed serum levels by 57.84%. At the maximum NNC dosage (50 mg/kg), trace metal accumulation peaked in all tissues. In muscle tissue, concentrations at 50 mg/kg NNC were 7.88 ± 0.20 µg/g (Zn), 8.56 ± 0.18 µg/g (Cu), and 8.02 ± 0.21 µg/g (Fe), whereas in the control group, the values were 4.74 ± 0.16 µg/g, 5.27 ± 0.14 µg/g, and 5.06 ± 0.17 µg/g, respectively. In serum, the levels of Zn, Cu, and Fe were 5.91 ± 0.13, 6.48 ± 0.15, and 6.09 ± 0.11 µg/g at a dosage of 50 mg/kg NNC, in contrast to 3.14 ± 0.11, 3.64 ± 0.09, and 3.68 ± 0.12 µg/g in the control group. Cu demonstrated the highest accumulation among the three trace metals in all tissues. Cu levels were, on average, 11.15% elevated compared to Zn and 1.09% elevated compared to Fe. Accumulation patterns exhibited a distinct dose-dependent trend, with trace element levels progressively increasing alongside elevated NNC concentration.

## 4. Discussion

This study examined the impact of a NNC including Zn, Cu, and Fe NPs on the growth performance, feed utilization efficiency, hematological profiles, and serum biochemistry of *Clarias batrachus*. Generally, the findings indicated that food supplementation with NNC markedly improved growth metrics, physiological well-being, and nutritional absorption, with an optimal dosage established at 30 mg/kg. These findings are consistent with those of Jewel et al. [6], who reported improved growth in *Labeo rohita* with 30 mg/kg of Fe–Zn NPs in a fish meal-based diet, while higher inclusion levels (40–50 mg/kg) led to diminished growth outcomes. In this study, however, at elevated inclusion levels of NNC (40 and 50 mg/kg), a decline in growth performance was observed, likely attributable to the toxic effects associated with excessive nanoparticle accumulation, indicating a threshold beyond which *C. batrachus* cannot efficiently utilize dietary NNC. A similar trend was reported by Jewel et al. [6], who documented reduced growth in *Labeo rohita* fed high concentrations of Fe–Zn NPs. Consistent findings were also noted by Faiz et al. [42], where juvenile grass carp (*Ctenopharyngodon idella*) exhibited diminished growth when fed 50 mg/kg ZnO NPs compared to those receiving 30 mg/kg. Similarly, Khan et al. [24] documented significantly enhanced growth in Nile tilapia fed 60 mg/kg of NNC compared to the control. The synergistic effects of ZnO and Se NPs on the growth of *L. rohita* were also demonstrated by Swain et al. [30]. Several additional studies corroborate the positive impact of nanoparticle-enriched diets on fish growth and health [20, 21, 42–44]. The underlying mechanisms by which NPs improve growth performance likely involve enhanced intestinal absorption, increased bioavailability of essential trace elements, stimulation of GH secretion, and elevated enzymatic and catalytic activities [45–47]. In the current experiment, no toxic effects of NNC were observed, as evidenced by a 100% SR of fish in all groups. Khan et al. [24] and Jewel et al. [6] also reported a 100% SR for Nile tilapia and Rohu in the NPs feed experiment. Based on the results obtained, it can be inferred that the consumption of NNC has no detrimental impacts on the overall health of fish. These findings collectively emphasize the critical need for precise dosing of nanoparticle supplements to maximize benefits while avoiding adverse effects.

Initial weights (IWs) were statistically uniform across all groups (5.21–5.29 g), confirming the homogeneity of starting fish populations (*p*  > 0.05), thus validating subsequent comparisons. Significant improvements in FW, %WG, ADG, and SGR were recorded in fish fed NNC at 20 and 30 mg/kg compared to the control and other treatment groups (*p*  < 0.05). The group receiving 30 mg/kg NNC exhibited the highest FW (25.73 ± 0.41 g), with WG increasing by approximately 80% compared to the control (386.67% vs. 215.33%). Similarly, ADG and SGR peaked in the NNC30 group (0.34 g/day and 2.64%/day, respectively), underscoring enhanced growth kinetics driven by the optimized nano-nutrient dose. These parameters declined significantly at higher NNC levels (40 and 50 mg/kg), indicating a dose threshold beyond which the NPs potentially exert toxic effects or metabolic burden, consistent with prior findings in other species [6, 42]. Notably, SRs remained at 100% across all treatments, including the highest NNC levels, indicating that despite the observed growth and feed utilization declines at elevated doses, no acute toxicity or mortality occurred within the 60-day period. This survival consistency aligns with prior nanoparticle feeding studies [6, 24] and supports the general safety of NNC within the tested range.

The CF, a proxy for fish health and robustness, also followed a similar trend, reaching the highest values at 30 mg/kg NNC (1.67 ± 0.15), significantly better than the control and other groups (*p* = 0.01). This suggests an overall improvement in body condition and nutrient assimilation efficiency at optimal NNC dosing. Feed utilization metrics further reinforce the benefits of NNC supplementation. The FCR was significantly reduced at 20 and 30 mg/kg NNC (1.42 and 1.24, respectively), reflecting more efficient feed utilization and reduced waste. In contrast, FCR increased markedly at 40 and 50 mg/kg (2.47 and 3.14), implying diminished feed efficiency and possible physiological stress at higher doses. The enhanced FCR observed in this study is consistent with previous reports demonstrating the beneficial effects of nanoparticle supplementation on feed efficiency. Ahmed et al. [48] reported improved FCR in fish fed NP-supplemented diets, likely due to accelerated growth rates. Similarly, Jewel et al. [6] documented dose-dependent improvements in feeding efficiency in *Labeo rohita* with Fe–Zn NP inclusion. These findings support the potential of NNC to enhance feed utilization efficiency in catfish aquaculture.

The PER and PGR mirrored this pattern, with maximum values at 30 mg/kg (2.44% and 1.60%, respectively), indicating superior protein utilization for growth. ANPU, representing the long-term capacity for dietary protein assimilation, was significantly enhanced in the NNC30 group (39.70%), nearly doubling that of the control (21.32%). Such improvement has direct implications for sustainable aquaculture, as it suggests that nano-formulated mineral supplementation can markedly increase nutrient conversion efficiency, reduce feed costs, and improve biomass yield.

Significnat improvements in the proximate composition of muscle—specifically elevated crude protein and lipid contents—highlighted the nutritional advantages of nanonutrient supplementation. These enhancements were dose-dependent and consistent with previous findings by Jewel et al. [6] and Muralisankar et al. [49], who observed similar trends in *Labeo rohita* and *Macrobrachium rosenbergii*, respectively, following dietary inclusion of Fe and Zn NPs. Mechanistically, these changes may be attributed to the activation of nutrient-sensitive pathways such as the mechanistic target of rapamycin (mTOR), which promotes protein synthesis and lean tissue accretion [50]. Concurrently, the observed reduction in muscle moisture content suggests an increase in nutrient density, a desirable trait in aquaculture products. However, as noted by Muralisankar et al. [49], excessive nanoparticle inclusion (e.g., 80 mg/kg Zn-NPs) may adversely affect muscle composition, indicating the importance of optimizing dosage levels.

Hematological profiles served as sensitive indicators of the physiological responses of *Clarias batrachus* to NNC supplementation. A clear dose-dependent enhancement in RBC count, Hb concentration, Hct, and related indices was observed up to 30 mg/kg, reflecting improved erythropoiesis and oxygen-carrying capacity, likely facilitated by the bioavailable iron content in the NNC [31]. These findings are consistent with the notion that hematological metrics are reliable markers of fish health, reflecting both nutritional status and environmental adaptability [51, 52]. However, at higher NNC inclusion (50 mg/kg), marked reductions in RBC, Hb, and Hct levels, alongside elevated WBC counts, were noted—indicative of systemic stress and possible hematotoxicity. This shift may stem from oxidative stress triggered by excess metal NPs, which are known to induce reactive oxygen species (ROS) and compromise cellular membrane integrity [53]. Comparable hematological disturbances have been documented in *Oreochromis niloticus* exposed to ZnO NPs, where reductions in erythrocyte indices suggested anemia and disrupted hematopoiesis [54]. Moreover, increased leukocyte counts at higher nanoparticle doses may reflect immunomodulatory responses or inflammation, as reported by Vani et al. [55] and Bantu et al. [56]. These patterns support earlier observations by Saravanan et al. [57] and Suganthi et al. [58], who emphasized the dual role of NPs in enhancing hematological performance at optimal levels, while eliciting toxicological responses at excessive dosages. Collectively, the data underscore the necessity of precise dose optimization to maximize physiological benefits while minimizing adverse hematological effects.

Further, serum biochemical analyses provided a useful insights into the physiological responses of *Clarias batrachus* to NNC supplementation. At the optimal dose (30 mg/kg), significant increases in total protein, albumin, and globulin levels were observed, indicating enhanced metabolic activity and immune function, consistent with findings by Kumar et al. [59]. These improvements highlight the antioxidant potential of NNC, as also noted by Mahboub et al. [60] in *Cyprinus carpio* exposed to ZnO NPs. However, supplementation at 50 mg/kg induced a notable elevation in liver enzymes (ALT and AST) and disrupted lipid profiles, suggesting hepatic stress and impaired lipid metabolism—hallmarks of nanoparticle-induced toxicity [23, 61]. The concurrent rise in serum biochemical markers at this higher dose may reflect an overactivated immune response, a condition often linked to physiological stress and increased disease susceptibility [61]. These results collectively emphsized the dual role of NNC as both a functional nutrient and a potential stressor at supraoptimal levels, emphasizing the importance of dose regulation to avoid subclinical toxicity. In this study, dietary supplementation with NNC containing Zn, Cu, and Fe was maintained within established nutritional safety thresholds based on NRC [34] guidelines and species-specific studies [6, 30, 32]. Zn (30–50 mg/kg), Cu (3–5 mg/kg), and Fe (30–70 mg/kg) levels were carefully optimized to avoid toxicity while enhancing growth, feed efficiency, and hematological and biochemical health parameters. No signs of clinical or subclinical toxicity were observed, confirming the biological safety of the dosing strategy. However, significant hepatic accumulation of trace elements—especially Cu—was noted at the highest dose (50 mg/kg), with the liver serving as the primary site of deposition, consistent with its detoxification role [62, 63]. These findings align with previous reports in *O. niloticus* [18, 41] and highlight the importance of long-term monitoring to prevent potential risks associated with nanoparticle bioaccumulation [64].

Collectively, the regression-derived optimal NNC dosage range (30.19–30.26 mg/kg) provides a robust, evidence-based guideline for improving growth and feed efficiency in *C. batrachus*. This precision supplementation strategy holds promise for addressing key challenges in sustainable aquaculture by enhancing nutrient utilization, reducing feed waste, and improving fish health, thereby contributing to improved production economics and environmental stewardship.

## 5. Conclusion

In conclusion, this study demonstrated that incorporating NNC in Asian catfish diets significantly enhances growth performance parameters, including SR, SGR, and FCR, while also improving key physiological indicators such as Hb, WBC count, enzymes, and mineral levels. Dietary NNC levels between 30.19 and 30.26 mg/kg were identified as optimal, showing marked improvements in FW, %WG, and SGR. Additionally, a 30 mg/kg NNC supplementation significantly increased protein and lipid content in the fish. However, higher doses (40–50 mg/kg) proved detrimental. Therefore, a diet containing 30 mg/kg of NNC is recommended as an optimal supplement for improving the growth and physiological health of *Clarias batrachus*, supporting sustainable and effective aquaculture practices.

## Figures and Tables

**Table 1:** Formulation and proximate composition of the basal diet*⁣*^*∗*^ used in feeding trials with *Clarias batrachus*.

Ingredients	g/kg	Proximate composition	%	
Fish meal^a^	275	Protein	32.88 ± 0.20	
Mustard oil cake^a^	200	Lipid	8.75 ± 0.13	
Soybean meal^a^	125	Moisture	8.17 ± 0.02	
Maize bran^a^	125	Ash	10.25 ± 0.20	
Wheat bran^a^	90	—	—	
Rice bran^a^	90	—	—	
Soybean oil^a^	60	—	—	
Choline chlorid^a^	2.5	—	—	
Zn-free premix^b^	32.5	—	—	

*Note*: Ingredients were locally sourced from Rajshahi, Bangladesh. Values are presented as mean ± SEM (*n* = 3).

^a^Ingredients purchased from local market of Rajshahi, Bangladesh.

^b^Zn, Cu, and Fe-free premix (mg/kg of premix): vitamin A, 156,000 IU; vitamin D3, 31,200 IU; vitamin E, 299; vitamin K3, 26; vitamin B1, 32.5; vitamin B2, 65; vitamin B6, 520; vitamin B12, 0.16; nicotinic acid, 520; folic acid, 10.4; copper, 130; iodine, 5.2; manganese, 780; selenium, 1.95. Premix was supplied by Reneta Animal Health Pharma Co. Ltd. Bangladesh.

*⁣* ^*∗*^Zn-NPs, Cu-NPs, and Fe-NPs were combined at a ratio of 40:20:40 and were added to the basal diet at 10, 20, 30, 40, and 50 mg/kg diet.

**Table 2:** Water quality parameters (mean ± SEM) in experimental tanks stocked with *Clarias batrachus* during a 60-day feeding trial with graded levels of dietary nanonutrient complex (NNC).

Parameters	Dietary NNC supplementation level in feed						*F*-value	*p*-Value
	Control	NNC10	NNC20	NNC30	NNC40	NNC50		
Temperature (°C)	27.89 ± 0.30^a^	28.30 ± 0.15^a^	28.20 ± 0.15^a^	28.75 ± 0.22^a^	28.25 ± 0.20^a^	28.18 ± 0.10^a^	3.54	0.77
DO (mg/L)	5.35 ± 0.22^a^	5.40 ± 0.25^a^	5.07 ± 0.17^a^	5.30 ± 0.36^a^	5.44 ± 0.33^a^	5.26 ± 0.12^a^	1.52	0.43
pH	7.20 ± 0.20^a^	7.50 ± 0.24^a^	7.30 ± 0.24^a^	7.30 ± 0.23^a^	7.19 ± 0.20^a^	7.25 ± 0.11^a^	10.82	0.12
Ammonia (mg/L)	0.002 ± 0.001^a^	0.003 ± 0.001^a^	0.002 ± 0.001^a^	0.002 ± 0.001^a^	0.001 ± 0.001^a^	0.002 ± 0.001^a^	0.82	0.30

*Note:* Values in the same row having same superscript letter indicates no significant difference (*p*  > 0.05). No significant differences (*p*  > 0.05) were observed across treatments.

Abbreviations: DO, dissolved oxygen; NNC, nanonutrient complex.

**Table 3:** Growth performance and feed utilization parameters (mean ± SEM) of *Clarias batrachus* fed diets containing different concentrations of nanonutrient complex (NNC) for 60 days.

Parameters	Dietary NNC supplementation level in feed						*F*-value	*p*-Value
	Control	NNC10	NNC20	NNC30	NNC40	NNC50		
IW (g)	5.21 ± 0.01^a^	5.22 ± 0.06^a^	5.21 ± 0.03^a^	5.29 ± 0.05^a^	5.23 ± 0.12^a^	5.25 ± 0.06^a^	0.72	0.62
FW (g)	16.43 ± 1.33^d^	19.46 ± 0.99^c^	22.89 ± 1.53^b^	25.73 ± 0.41^a^	15.48 ± 1.01^de^	13.47 ± 1.61^e^	44.63	0.001
%WG	215.33 ± 25.77^d^	273.00 ± 19.31^c^	339.67 ± 28.18^b^	386.67 ± 10.12^a^	196.33 ± 25.11^de^	157.00 ± 31.05^e^	40.00	0.001
ADG (g)	0.19 ± 0.02^d^	0.24 ± 0.02^c^	0.30 ± 0.03^b^	0.34 ± 0.01^a^	0.17 ± 0.02^de^	0.14 ± 0.03^e^	40.50	0.001
SGR (%/day)	1.91 ± 0.13^c^	2.20 ± 0.09^b^	2.46 ± 0.11^a^	2.64 ± 0.03^a^	1.80 ± 0.14^c^	1.56 ± 0.21^d^	30.32	0.001
CF	1.19 ± 0.14^c^	1.22 ± 0.27^c^	1.59 ± 0.11^b^	1.67 ± 0.15^a^	1.22 ± 0.19^c^	1.15 ± 0.09^c^	5.42	0.01
SR (%)	100	100	100	100	100	100	—	—
FCR	2.25 ± 0.26^bc^	1.76 ± 0.12^cd^	1.42 ± 0.12^d^	1.24 ± 0.03^d^	2.47 ± 0.31^b^	3.14 ± 0.63^a^	15.65	0.001
PER	1.36 ± 0.16^d^	1.73 ± 0.12^c^	2.14 ± 0.18^b^	2.44 ± 0.06^a^	1.24 ± 0.16^de^	0.99 ± 0.20^e^	40.32	0.001
PGR (%)	1.37 ± 0.01^cd^	1.47 ± 0.07^bc^	1.58 ± 0.08^ab^	1.60 ± 0.10^ab^	1.69 ± 0.10^a^	1.26 ± 0.15^d^	8.92	0.001
ANPU (%)	21.32 ± 1.90^d^	27.50 ± 0.74^c^	35.08 ± 2.60^b^	39.70 ± 3.03^a^	24.31 ± 2.17^cd^	15.56 ± 3.82^e^	36.30	0.001

*Note:* Values with different superscript letters in the same column indicate significant difference at *p*  < 0.05. Superscript letters indicate significant differences (*p*  < 0.05) among treatments within rows. SGR, specific growth rate of weight.

Abbreviations: ADG, average daily gain of weight; ANPU, annual net protein utilization; CF, condition factor; FCR, feed conversion ratio; FW, final weight; IW, initial weight; NNC, nanonutrient complex; PER, protein efficiency ratio; PGR, protein growth rate; SR, survival rate; WG, weight gain; %WG, percentage weight gain.

**Table 4:** Hematological parameters (mean ± SEM) of *Clarias batrachus* fed different levels of dietary nanonutrient complex (NNC) for 60 days.

Parameters	Dietary NNC supplementation level in feed						*F*-value	*p*-Value
	Control	NNC10	NNC20	NNC30	NNC40	NNC50		
RBC (10^6^/mm^3^)	2.88 ± 0.02^c^	3.05 ± 0.06^c^	3.29 ± 0.08^b^	3.62 ± 0.21^a^	2.85 ± 0.11^c^	2.31 ± 0.14^d^	42.87	0.001
WBC (10^6^/mm^3^)	6.69 ± 0.22^d^	6.92 ± 0.16^cd^	7.28 ± 0.08^c^	7.28 ± 0.11^c^	7.79 ± 0.18^b^	8.77 ± 0.34^a^	42.18	0.001
Hemoglobin (g/dL)	9.31 ± 0.10^ab^	9.34 ± 0.36^ab^	9.63 ± 0.02^b^	9.83 ± 0.12^a^	8.98 ± 0.20^b^	6.82 ± 0.62^c^	37.41	0.001
Hematocrit (%)	25.17 ± 0.92^a^	25.52 ± 1.00^a^	25.70 ± 0.99^a^	26.31 ± 0.52^a^	22.03 ± 1.85^b^	17.85 ± 0.84^c^	26.55	0.001
MCV (fL)	72.86 ± 4.96^c^	78.18 ± 4.76^bc^	83.69 ± 3.95^ab^	87.52 ± 3.62^a^	77.31 ± 7.62^bc^	77.42 ± 1.16^bc^	3.64	0.03
MCH (pg/cells)	27.19 ± 1.24^c^	29.29 ± 0.77^bc^	30.52 ± 0.66^ab^	32.46 ± 1.21^a^	31.51 ± 1.78^ab^	29.55 ± 1.25^b^	7.05	0.001
MCHC (%)	3.74 ± 0.11^a^	3.75 ± 0.14^a^	3.65 ± 0.11^a^	3.72 ± 0.25^a^	4.09 ± 0.27^a^	3.82 ± 0.19^a^	2.06	0.14

*Note:* Superscript letters indicate significant differences (*p*  < 0.05) within columns.

Abbreviations: MCH, mean corpuscular hemoglobin; MCHC, mean corpuscular hemoglobin concentration; MCV, mean corpuscularvolume; NNC, nanonutrient complex; RBC, red blood cells; WBC, white blood cells.

**Table 5:** Serum biochemical profile (mean ± SEM) of *Clarias batrachus* after a 60-day feeding trial with various levels of dietary nanonutrient complex (NNC).

Parameters	Dietary NNC supplementation level in feed						*F*-value	*p*-Value
	Control	NNC10	NNC20	NNC30	NNC40	NNC50		
Total protein (g/dL)	3.14 ± 0.06^e^	4.57 ± 0.09^d^	4.68 ± 0.03^c^	4.79 ± 0.05^b^	4.88 ± 0.02^ab^	4.95 ± 0.05^a^	504.49	0.001
Albumin (g/dL)	0.47 ± 0.02^e^	1.47 ± 0.06^cd^	1.41 ± 0.04^d^	1.55 ± 0.04^bc^	1.59 ± 0.06^ab^	0.98 ± 0.03^a^	279.99	0.001
Globulin (g/dL)	2.66 ± 0.04^d^	3.10 ± 0.10^c^	3.27 ± 0.04^b^	3.24 ± 0.02^b^	3.29 ± 0.03^b^	3.97 ± 0.04^a^	214.01	0.001
Total cholesterol (mg/dL)	212.72 ± 2.48^bc^	209.58 ± 2.80^c^	207.87 ± 2.24^c^	193.71 ± 4.87^d^	217.12 ± 1.41^b^	224.84 ± 1.10^a^	42.54	0.001
HDL (mg/dL)	53.29 ± 0.83^bc^	53.72 ± 1.35 ^bc^	52.62 ± 1.16^ab^	52.28 ± 0.34^c^	54.00 ± 0.52^abc^	55.75 ± 1.13^a^	4.54	0.02
LDL (mg/dL)	150.99 ± 1.23^c^	151.36 ± 0.60^bc^	152.27 ± 1.14^bc^	152.47 ± 0.77^bc^	152.93 ± 0.56^b^	154.83 ± 0.82^a^	7.02	0.001
Triglycerides(mg/dL)	138.53 ± 3.36^b^	142.26 ± 4.29^a^	156.30 ± 3.53^a^	157.41 ± 4.34^a^	159.29 ± 3.63^a^	159.80 ± 3.07^a^	14.25	0.001
ALP (mg/dL)	7.85 ± 1.03^c^	8.73 ± 0.72^c^	9.28 ± 0.65^c^	11.87 ± 1.15^b^	13.32 ± 1.51^b^	17.08 ± 2.37^a^	19.35	0.001
ALT (U/L)	28.47 ± 0.85^c^	30.22 ± 0.97^b^	30.82 ± 1.19^b^	31.26 ± 0.69^b^	31.65 ± 0.20^b^	33.75 ± 0.39^a^	14.50	0.001
AST (U/L)	34.04 ± 0.50^d^	34.65 ± 0.26^cd^	34.93 ± 0.17^cd^	35.47 ± 1.01^c^	37.25 ± 0.41^b^	40.29 ± 1.17^a^	33.60	0.001
Amylase (U/L)	0.31 ± 0.02^e^	0.49 ± 0.03^d^	0.66 ± 0.10^c^	0.74 ± 0.13^bc^	0.84 ± 0.03^b^	1.80 ± 0.11^a^	121.62	0.001
Lipase (U/L)	0.27 ± 0.03^e^	0.29 ± 0.03d^e^	0.36 ± 0.05^cd^	0.44 ± 0.04^c^	0.56 ± 0.02^b^	1.85 ± 0.07^a^	583.78	0.001
Protease (U/L)	0.66 ± 0.04^d^	0.66 ± 0.12^d^	0.76 ± 0.09^cd^	0.86 ± 0.03^bc^	0.94 ± 0.02^b^	1.51 ± 0.06^a^	61.61	0.001

*Note:* Different superscript letters indicate significant differences (*p*  < 0.05) across treatments for each parameter.

Abbreviations: ALP, alkaline phosphatase; ALT, alanine aminotransferase; AST, aspartate aminotransferase; HDL, high-density lipoprotein; LDL, low-density lipoprotein.

## Data Availability

The data will be made available upon request.
